# Prediction Models for the Content of Calcium, Boron and Potassium in the Fruit of ‘Huangguan’ Pears Established by Using Near-Infrared Spectroscopy

**DOI:** 10.3390/foods11223642

**Published:** 2022-11-14

**Authors:** Jing Fang, Xiu Jin, Lin Wu, Yuxin Zhang, Bing Jia, Zhenfeng Ye, Wei Heng, Li Liu

**Affiliations:** 1 School of Horticulture, Anhui Agricultural University, 130 Changjiang West Road, Hefei 230036, China; 2 School of Information and Computer Science, Anhui Agriculture University, 130 Changjiang West Road, Hefei 230036, China

**Keywords:** ‘Huangguan’ pear, mineral element, spectral analysis, modelling, content prediction

## Abstract

It has been proved that the imbalance of the proportion of elements of ‘Huangguan’ pears in the pulp and peel, especially calcium, boron and potassium, may be important factors that can seriously affect the pears’ appearance quality and economic benefits. The objective of this study was to predict the content of calcium, boron and potassium in the pulp and peel of ‘Huangguan’ pears nondestructively and conveniently by using near-infrared spectroscopy (900–1700 nm) technology. Firstly, 12 algorithms were used to preprocess the original spectral data. Then, based on the original and preprocessed spectral data, full-band prediction models were established by using Partial Least Squares Regression and Gradient Boosting Regression Tree. Finally, the characteristic wavelengths were extracted by Genetic Algorithms to establish the characteristic wavelength prediction models. According to the prediction results, the value of the determination coefficient of the prediction sets of the best prediction models for the three elements all reached ideal levels, and the values of their Relative analysis error also showed high levels. Therefore, the micro near-infrared spectrometer based on machine learning can predict the content of calcium, boron and potassium in the pulp and peel of ‘Huangguan’ pears accurately and quickly. The results also provide an important scientific theoretical basis for further research on the degradation of the quality of ‘Huangguan’ pears caused by a lack of nutrients.

## 1. Introduction

‘Huangguan’ pears (*Pyrus bretschneideri* Rehd cv.Huangguan), which are famous for their excellent quality in Chinese and even the world’s pear market, have been introduced and planted in many places with good performance and have a good development prospect [1,2]. In actual production, ‘Huangguan’ pears are often bagged to prevent pests and diseases. However, brown spots that often appear on the surface of bagged ‘Huangguan’ pears have been seriously affecting the appearance quality and economic benefits of the fruits [3,4]. A large number of studies have proved that the content of calcium, boron and potassium may be important factors affecting the appearance quality of bagged ‘Huangguan’ pears [5,6,7,8,9]. The traditional detection of the mineral nutrient content is mostly based on laboratory physical and chemical analysis, including inductively coupled plasma mass spectrometry (ICP-MS), atomic absorption spectrometry, UV-visible spectrophotometer measurement and so on [10,11,12]. Although the results are accurate, destructive sampling, high time consumption, high labor intensity, high costs and some other disadvantages have brought many limitations to the study of pear fruit mineral nutrition. If the mineral nutrient content in the fruit of pears can be conveniently and nondestructively detected by modern technology during the critical period of the growth and development of ‘Huangguan’ pears, then the fertilization and other measures can be adjusted accordingly, and it will be of great significance to improve the quality of the fruit of ‘Huangguan’ pears.

Up to now, some methods are often used for the nondestructive testing of fruits, such as hyperspectral imaging technology, near-infrared spectroscopy, X-ray testing, etc. [13]. Among them, the near-infrared spectroscopy detection method is favored by many researchers because of its advantages of nondestructiveness, convenience, environmental protection and safety. The absorption of molecules within the coverage of the near-infrared spectrum consists of the frequency combination and double frequency absorption of hydrogen-containing groups or other chemical bonds in various organic substances, mainly including C-H, N-H, S-H, O-H, etc. Therefore, the spectral curve obtained by using near-infrared spectroscopy technology can better reflect the information of organic substances containing hydrogen groups in the tested samples and composition information in various other biochemical structures [14].

At present, near-infrared spectroscopy is applied to the detection and research of fruit quality, for example, the internal dry matter content (DMC) and soluble solid concentration (SSC) of peach fruit, and the prediction of the titratable acidity, pH value and maturity index in citrus [15,16,17,18,19,20,21,22]. Many investigators have also used near-infrared spectroscopy to predict the mineral content of different trees or crops. Wiebke et al. [23] quickly measured the content of calcium and boron in Hass avocado fruit with near-infrared hyperspectral imaging, greatly improving the previous time-consuming and destructive laboratory method for evaluating fruit quality. Cozzolino et al. [24] explored the use of near-infrared spectroscopy to predict the electrical conductivity and elements Ca, Mg, K, etc. in grapes as a fast method to monitor such component parameters. Wang et al. [25] used the visible-near-infrared spectroscopy technique combined with the Partial Least Squares Regression method to establish a full-band (350–2500 nm) pear leaf nitrogen content prediction model more accurately, and based on this, timely estimated the nitrogen content in supplementary topdressing; Damián [26] et al. used near-infrared spectroscopy combined with MPLS to determine the content of calcium, potassium, magnesium and other mineral elements in zucchini, and promoted summer squash varieties rich in micronutrients; Rossa et al. [27] used near-infrared spectroscopy to determine some mineral nutrients in mate tea leaves and found that the NIRS had a good effect on predicting the contents of phosphorus, copper and iron in leaves, but it was not effective in the prediction of sodium, zinc and manganese and potassium content. It can be seen that NIRS technology has great potential in the determination of the mineral nutrient content of pear fruit. Although near-infrared spectroscopy is more suitable for detecting organic compounds, it can still reflect the information of inorganic parts by indirectly measuring other compounds that can be directly measured by near-infrared spectroscopy, or by combining inorganic compounds with organic compound functional groups or directly with organic matrix.

Therefore, ‘Huangguan’ pears were taken as the research object to explore the effect of using near-infrared spectroscopy (900–1700 nm) technology to predict the content of calcium, boron and potassium in pear fruit in this paper. Firstly, based on the original spectral data and the spectral data obtained after pretreatment, and successive uses of PLSR and GBRT to establish full-band models for predicting the content of the mineral elements calcium, boron and potassium in the pulp and peel of ‘Huangguan’ pears, we used a genetic algorithm to extract the characteristic wavelengths and establish characteristic wavelength models. According to the model evaluation index-determination coefficient and Relative analysis error, the best models for predicting the content of mineral elements were selected to provide some theoretical basis for the nondestructive detection of the mineral nutrient content of pear fruits.

## 2. Materials and Methods

### 2.1. Samples

The tested pear fruits were from a horticultural farm located in Dangshan County, Anhui Province, which is known as the ‘pear capital’ of China. In mid-August 2021, at 26 °C, the fruits were picked and transported back to the laboratory by cartons with good air permeability. A total of 65 ‘Huangguan’ pears with uniform appearance and no damage were strictly selected as the test samples. The surface of the pears was washed, then wiped clean and numbered for further use.

### 2.2. Near-Infrared Spectrum Data Acquisition

**Figure 1:**
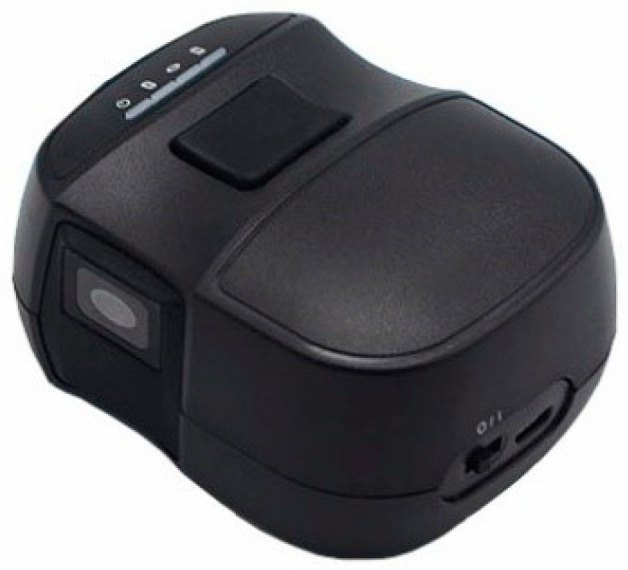
‘NIR-S-G1′ miniature spectrum acquisition instrument.

The near-infrared spectrum acquisition instrument used was a reflective handheld near-infrared spectrometer with the model ‘NIR-S-G1′ produced by the Shenzhen Ugreen Group, Ltd. (Figure 1) (Shenzhen, China). The detected spectral wavelength range was from 900 nm to 1700 nm with 228 bands of spectrum acquisition points on it. The spectral resolution was 3.89 nm, and the signal-to-noise ratio (SNR) was 5000:1.

Before each measurement, the spectrometer was calibrated with a black and white plate. The material used for the white board calibration was polytetrafluoroethylene. The instrument was closely attached to the calibration white board. The light emitted by the instrument shined on the white board and reflected. This reflected light was captured and recorded as the brightness value (W) of the white board; for the blackboard correction, we turned off the emission light source of the instrument and recorded the brightness value captured by the instrument when there was no light source, which was recorded as the blackboard brightness value (B). After calibration, the instrument was used to measure the spectral data of the pear surface, the light source window on the instrument was close to the pear sample and the reflected light obtained was recorded as the brightness value (R) of the pear surface. The spectral reflectance of the sample can be calculated by Formula (1). The app ‘Instagram’ on a mobile phone was connected with the instrument by Bluetooth before use, and then the spectral data was collected.

R = (I − B)/(W − B)(1)

**Figure 2:**
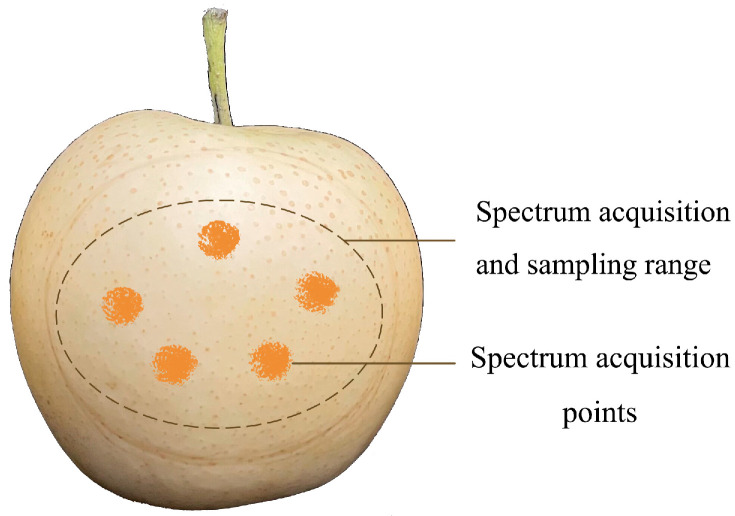
Schematic diagram showing the acquisition points for spectral sampling.

In the spectral measurement process, it is inevitable that the results will be affected by environmental factors. If the data are collected only once, the error will be too large. Therefore, in order to make the measurement results as accurate as possible, we took five points on the surface of each pear sample and used each point to collect spectral data once, and we repeated this five times. The specific operations were as follows: As shown in Figure 2, before collecting spectral data, an ellipse with a short axis length of about 5 cm and a long axis length of about 8 cm was delimited near the equator of the surface of each pear, and the area within the ellipse was used as the scope of subsequent spectral data collection and sampling. Then, we closed the scanning window at the front end of the spectrometer to the designated range on the surface of pears, scanned once at each spectral acquisition point and scanned 5 times for each pear sample in total. Finally, the average of the reflectance spectral data of the 5 scans of each pear sample was taken as the original modeling spectral data.

### 2.3. Detection of Mineral Elements in the Fruit of Pears

After the spectral data collection was completed, the pulp and peel of each pear were sampled within the designated range, packed in self-sealing bags and marked with numbers. The content of the mineral elements calcium, boron and potassium in the samples were detected by the instruments in the Anhui Institute of geological experiments. The detection basis was GB 5009.268-2016, and the main instrument used was an inductively coupled plasma emission spectrometer/B1497.

### 2.4. Near-Infrared Spectrum Data Information Preprocessing and Characteristic Wavelength Extraction

Since there is no literature indicating which preprocessing method is the best, trial and error is usually used to determine the appropriate spectral data preprocessing method [28]. Therefore, after obtaining the original spectral curve based on the algorithms first derivative (FD), second derivative (SD), Multiple Scattering Correction (MSC), Standard Normal Variate (SNV), Savitzky Golay Convolution Smoothing (SG) and Logarithmic Transformation (LG), we used algorithms combined by two single preprocessed methods—SG+MSC and SG+SNV—and algorithms combined by three single preprocessed methods—SG+MSC+SD, SG+MSC+FD, SG+SNV+FD and SG+SNV+SD—to preprocess the original spectral data and compare the impact of different preprocessed methods on the later modeling accuracy.

After preprocessing based on Genetic Algorithms (GA), we extracted the characteristic wavelengths of the full-band models that met the prediction standard, and then established the characteristic wavelength models to further optimize the models [29,30]. 

### 2.5. Establishment of Prediction Models for the Content of Mineral Elements in the Fruit of ‘Huangguan’ Pears

Partial Least Square Regression (PLSR) is a classical statistical method; it combines the advantages of principal component analysis, multiple linear regression, and canonical correlation analysis, and can solve the problems of spectral data redundancy and collinearity [31,32]. It can be seen that PLSR is superior to the general least squares method, so we selected PLSR as one of the modeling methods. Gradient Boosting Regression Tree (GBRT) uses the boosting integration strategy to integrate and improve the traditional algorithms. It combines the advantages of multiple algorithms and has great advantages in nonlinear fitting. However, in the process of model training, it is necessary to continuously adjust the parameters to achieve a more reasonable training effect [33]. Therefore, we also selected GBRT as another modeling method.

Additionally, the model evaluation indicators in this paper are the determination coefficient (R^2^) and Relative Percent Deviation (RPD). The value range of the determination coefficient (R^2^) is (0, 1). The closer R^2^ is to 1, the smaller the error is, the more stable the model is, and the better the prediction effect is. Relative analysis error (RPD) is an important indicator to evaluate the quality of the model. This paper evaluates the prediction abilities of the models according to the RPD evaluation grade proposed by Chang et al. [34]: when E_RPD_ ≥ 2, it indicates that the prediction effect of the model is very good, and the model belongs to level A, which can be applied to the corresponding quantitative prediction; when 1.4 ≤ E_RPD_ < 2, it indicates that the model has the moderate predictive ability and belongs to level B; when E_RPD_ < 1.4, it indicates that the prediction effect of the model is relatively poor, and the model belongs to level C and cannot be used for quantitative prediction.

## 3. Results

### 3.1. Analysis of Original Spectral Curve

**Figure 3:**
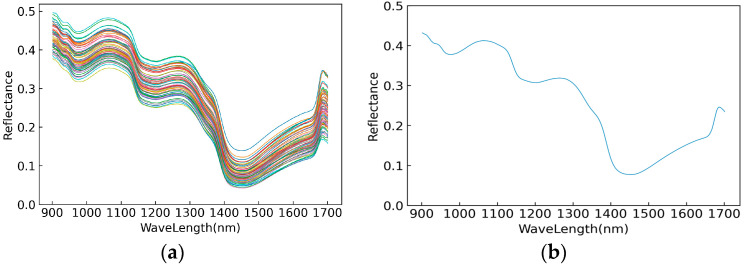
Original spectral reflectance map (**a**) and average spectral reflectance map (**b**) of all tested ‘Huangguan’ pears.

It can be seen from Figure 3 that the overall shape of the spectral curves of all ‘Huangguan’ pear fruit samples was similar, with clear peaks at wavelengths of 1050 nm, 1260 nm and 1680 nm, and clear troughs at 980 nm, 1200 nm, 1450 nm and 1660 nm. According to the literature, 980 nm and 1450 nm are the main water absorption peaks, corresponding to the first frequency doubling information of the O-H bond stretching vibration of related carbohydrates, and about 1660 nm corresponds to the first frequency doubling information of the C-H bond stretching vibration in methyl [35]. The main chemical components in pears include flavones, triterpenes, phenolic acids, luminol esters, polysaccharides, etc., of which polyphenols are the most abundant component, which are also very valuable secondary metabolites in pears, including chlorogenic acid, arbutin, coumaric acid, etc. [36]. At the same time, phenolic acids, flavones and triterpenes are usually more abundant in pear peels than that in pear pulp [37]. The presentation of the spectral curve, which corresponds to the frequency doubling and frequency information of different chemical bond vibrations accordingly, was complicated because of the complex compounds in pear fruits.

### 3.2. Analysis of Spectral Data Preprocessing Results

**Figure 4:**
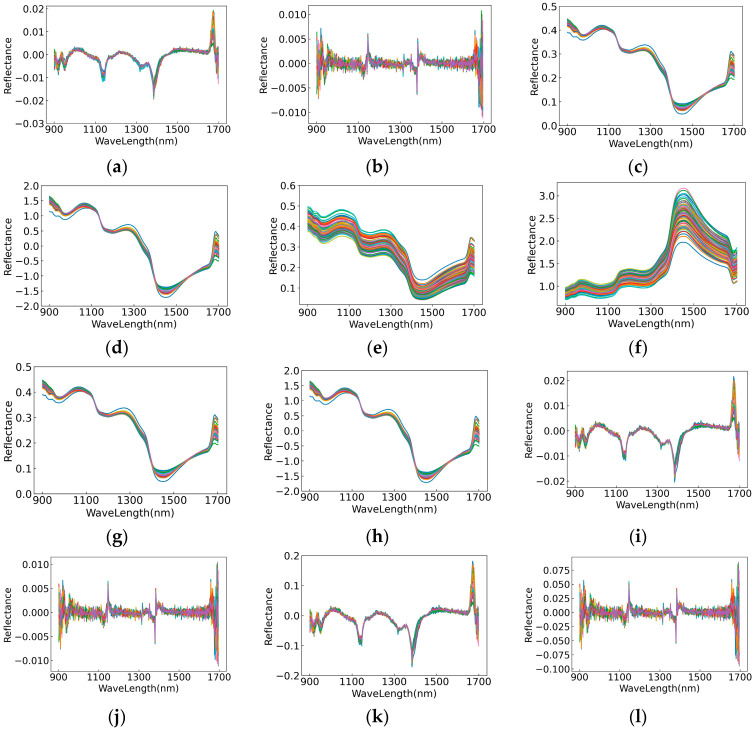
Spectral graphs after transformation based on different preprocessing methods. (**a**) FD, (**b**) SD, (**c**) MSC, (**d**) SNV, (**e**) SG, (**f**) LG, (**g**) SG+MSC, (**h**) SG+SNV, (**i**) SG+MSC+FD, (**j**) SG+MSC+SD, (**k**) SG+SNV+FD, (**l**) SG+SNV+SD.

The spectral curves obtained by twelve pretreatment methods are shown in Figure 4. The different pretreatment methods had different effects. Firstly, it can be seen from Figure 4a,b that the baseline offset phenomenon was improved to a certain extent after the derivative methods, and the signal-to-noise ratio also decreased, but the noise also increased accordingly. The processing of the second derivative algorithm had an even greater noise impact than the first derivative algorithm. Secondly, the spectral curves after being processed by MSC and SNV are shown in Figure 4c,d. MSC and SNV had no obvious tendency to change the original spectral curve. MSC transformation mainly eliminated the scattering effect caused by uneven particle distribution and different particle sizes on the surface of the samples, and SNV was mainly used to eliminate the influence of scattering and optical path changes on the spectrum. Thirdly, it can be seen from Figure 4e that the overall shape of the spectral curve preprocessed by SG was similar to the original spectral curve. SG removed some irrelevant noise based on the original spectral data, making the spectral curve smoother. Fourthly, LG reduced the absolute value of the spectral data to a certain extent, which was conducive to later calculation. In general, the curves processed by MSC, SNV, SG, SG+MSC and SG+SNV were similar to the original spectral curves. In addition, the curves pretreated by FD, SG+MSC+FD and SG+SNV+FD were similar, and the curves pretreated by SD, SG+MSC+SD and SG+MSC+SD were also similar.

### 3.3. Characteristic Analysis of Sample Detection Data

From the data shown in Table 1, the contents of calcium, boron and potassium in the peel were higher than those in the pulp, and the difference was obvious. In the pulp, the content of potassium was much higher than that of calcium and boron, and it was higher in the peel as well.

**Figure 5:**
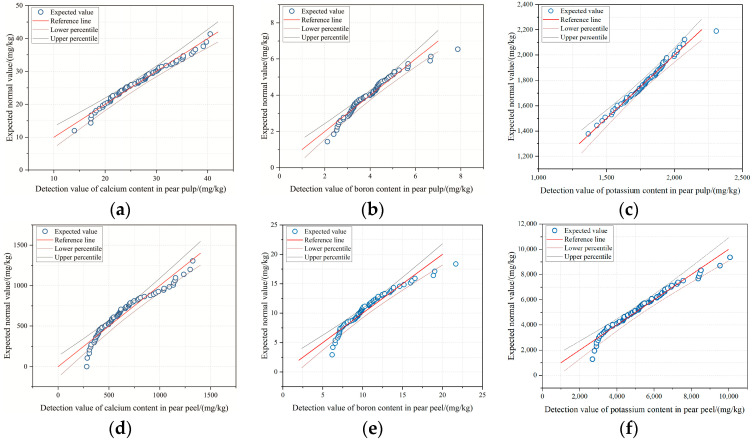
Curves fitted for prediction of mineral elements in pear pulp and peel. (**a**) Distribution of calcium content in pear pulp; (**b**) distribution of boron content in pear pulp; (**c**) distribution of potassium content in pear pulp; (**d**) distribution of calcium content in pear peel; (**e**) distribution of boron content in pear peel; (**f**) distribution of potassium content in pear peel.

It can be seen from Figure 5 that except for a few samples that were far away from the reference line, other samples were closely distributed around the reference line, where the *x*-axis value is the actual detection value and the corresponding *y*-axis value is the expected normal value.

### 3.4. Modelling and Prediction Analysis of the Content of Mineral Elements in Pear Pulp

Table 2 shows the comparison of parameters R^2^ and RPD of each prediction model for calcium, boron and potassium content in pear pulp based on full-wave band.

#### 3.4.1. Modelling and Prediction Analysis of the Content of Calcium in Pear Pulp

(1) Full-band based modelling

Based on the original spectral data and the spectral data after 12 kinds of pretreatment, 13 PLSR and GBRT models were established to predict the content of calcium in pear pulp. Among the 13 PLSR models, Raw-PLSR (prediction set R^2^ = 0.716, RPD = 1.431) and SG-PLSR (prediction set R^2^ = 0.717, RPD = 1.433) met level B. However, the predictive effects of the GBRT models for predicting the content of calcium in the pear pulp based on the full-wave band were not ideal, and they could not be used for the prediction of the content of calcium in pear pulp.

(2) Characteristic wavelength extraction

**Figure 6:**
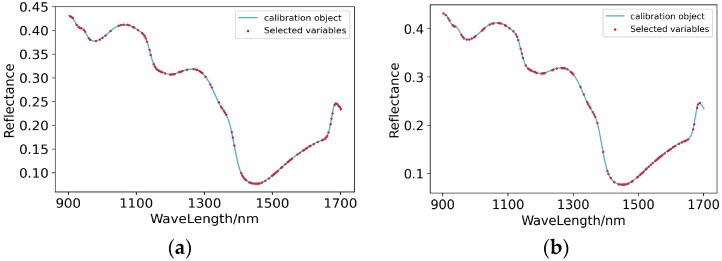
Characteristic wavelengths’ distribution of prediction models for the content of calcium in pear pulp. (**a**) Raw-GA-PLSR; (**b**) SG-GA-PLSR.

Based on GA, 125 and 119 characteristic wavelengths were extracted from the original spectral curves of RAW-PLSR and SG-PLSR, respectively. As shown in Figure 6, the characteristic wavelengths extracted from the original spectral curve points of Raw-GA-PLSR were concentrated at 1050–1300 nm and 1400–1680 nm, and at the same time, the characteristic wavelengths extracted from the original spectral curve points of SG-GA-PLSR were concentrated at 950–1300 nm and 1400–1680 nm. Although the specific values of the characteristic wavelengths extracted from the spectral curves of Raw-GA-PLSR and SG-GA-PLSR were not the same, their distribution ranges on their original spectral curves were similar, which indicates that the characteristic wavelength points corresponding to the compounds related to the content of calcium in pear pulp were roughly concentrated in the range above.

(3) Modelling of characteristic wavelengths

**Figure 7:**
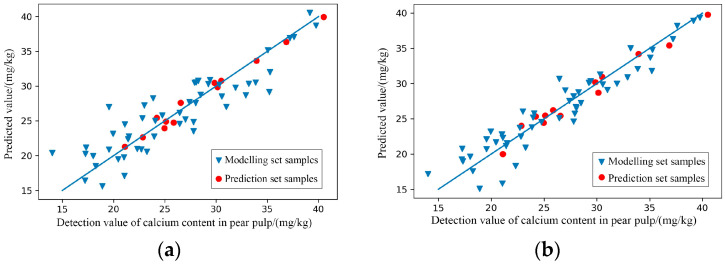
Scatter plots of models’ prediction results of the content of calcium in pear pulp samples. (**a**) Raw-GA-PLSR; (**b**) SG-GA-PLSR.

According to the modelling results presented in Table 3 and Figure 7, after GA treatment, the two models for predicting the content of calcium in pear pulp—Raw-PLSR and SG-PLSR—showed a slight ‘underfitting’ state (the effect of the prediction set was slightly better than that of the modelling set), which may have been due to the sample size, but the prediction performance was improved to level A. Furthermore, the prediction performance of the two models was better than before, which further shows that the feature extraction process can remove wavelengths that are not relevant to later modelling to a certain extent, and keep the features with rich information as much as possible to improve the predictive effect of the models.

(4) Modelling effect evaluation

After comparative analysis, among the established models for predicting the content of calcium in the pulp of ‘Huangguan’ pears, Raw-GA-PLSR showed the best prediction effect and met level A, whose modelling sets R^2^ and RPD were 0.987 and 6.110, respectively.

#### 3.4.2. Modelling and Prediction Analysis of the Content of Boron in Pear Pulp

(1) Full-band based modelling

A total of 65 samples of pear pulp boron content were randomly divided into a modeling set at 52 and a prediction set at 13, and then subsequent modeling analyses were carried out. Among the 13 PLSR models established, only RAW-PLSR and SG-PLSR met the prediction standards; moreover, the R^2^ of the modelling sets and prediction sets of SG-PLSR increased slightly and reached level B, and at the same time, the R^2^ of the modelling sets and prediction sets of the remaining 11 models decreased to varying degrees, among which SD-PLSR, SG+MSC+SD-PLSR and SG+SNV+SD-PLSR failed. Among the 13 GBRT models established, except LG-GBRT, the R^2^ and RPD of the modelling sets of the other 11 models based on pretreatment spectra were improved compared with RAW-PLSR. Although the R^2^ and RPD of the prediction sets of SNV-GBRT, MSC-GBRT, SD-GBRT, SG+MSC-GBRT, SG+SNV-GBRT and SG+MSC+FD-GBRT were improved compared with RAW-GBRT, they still did not meet the prediction standard.

(2) Characteristic wavelength extraction

**Figure 8:**
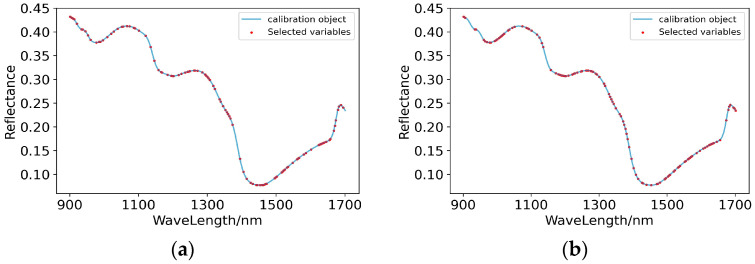
Characteristic wavelengths’ distribution of prediction models for the content of boron in pear pulp. (**a**) Raw-GA-PLSR; (**b**) SG-GA-PLSR.

Among the 26 models for predicting the content of boron in pear pulp based on the full-wave band, RAW-PLSR and SG-PLSR, which initially met the prediction standards, were extracted with characteristic wavelengths. The genetic algorithm extracted 116 and 118 characteristic wavelengths from the spectral curves of RAW-PLSR and SG-PLSR, respectively. As shown in Figure 8, in the original spectral curve points of Raw-GA-PLSR, the extracted characteristic wavelengths were concentrated at 900–1100 nm, 1200–1400 and 1500–1700 nm; meanwhile, in the original spectral curve points of SG-GA-PLSR, the extracted characteristic wavelength points were mainly distributed at 950–1100 nm and 1200–1680 nm. The dense distribution areas of the characteristic wavelengths of the two models were similar, indicating that the compounds related to the content of boron in pear pulp mainly responded in the range of 950–1100 nm, 1200–1400 nm and 1500–1680 nm.

(3) Modelling of characteristic wavelengths

**Figure 9:**
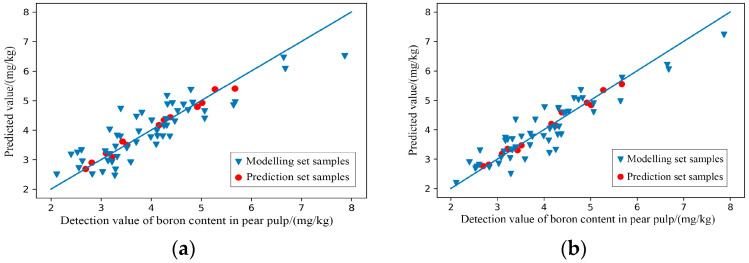
Scatter plots of models’ prediction results of the content of boron in pear pulp samples. (**a**) Raw-GA-PLSR; (**b**) SG-GA-PLSR.

After GA treatment, the predictive effects of RAW-PLSR and SG-PLSR for predicting the content of boron in pear pulp was significantly improved as the results shown in Table 4. The scatter plots of the prediction results shown in Figure 9 verify the good prediction effects of RAW-GA-PLSR and SG-GA-PLSR. The R^2^ of the two model prediction sets exceeded 0.9, reaching a relatively accurate prediction level, while the R^2^ of the modelling set was slightly lower than that of the respective prediction sets. Although it showed a slight ‘underfitting’ state, it did not affect the overall predictive effects.

(4) Modelling effect evaluation

After analysis and discussion, SG-GA-PLSR had the best effect on predicting the content of boron in pear pulp, whose R2 = 0.846 and RPD = 1.878 in the modelling set; R2 = 0.986 and RPD = 6.033 in the prediction set; and the effect of the prediction set had reached level A.

#### 3.4.3. Modelling and Prediction Analysis of the Content of Potassium in Pear Pulp

(1) Full-band based modelling

Among the 13 established PLSR models, Raw-PLSR and SG-PLSR met the predicting standards, while the fitting results of the remaining 11 PLSR models did not meet the expectations. While among the 13 established GBRT models, RAW-GBRT, SNV-GBRT, FD-GBRT, MSC-GBRT, SG+MSC-GBRT, SG+SNV-GBRT, SG+MSC+FD-GBRT and SG+SNV+FD-GBRT were invalid, SD-GBRT, SG-GBRT, LG-GBRT, SG+MSC+SD-GBRT and SG+SNV+SD-GBRT did not meet the prediction standards. On the whole, the established GBRT models were not suitable for the prediction of the content of potassium in ‘Huangguan’ pear pulp, and the reasons may be as follows: On one hand, GBRT is an additive model, and the next round is the residual between the predicted value and the actual value of the previous round, which is used as a label to continue fitting, and finally, the results are added, which in the end may lead to cases where R^2^ is negative or the prediction is invalid. On the other hand, the number of samples, data detection errors and so on may also be other reasons.

(2) Characteristic wavelength extraction

**Figure 10:**
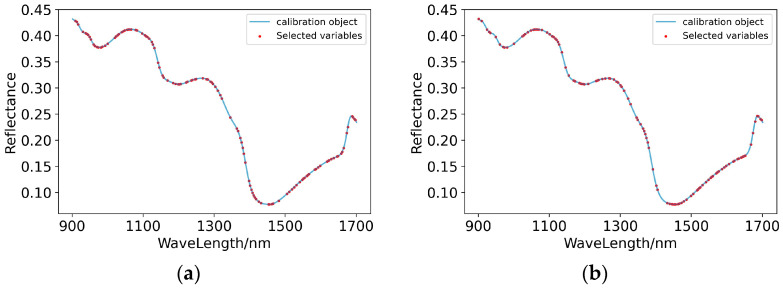
Characteristic wavelengths’ distribution of prediction models for the content of potassium in pear pulp. (**a**) Raw-GA-PLSR; (**b**) SG-GA-PLSR.

The spectral curves of Raw-PLSR and SG-PLSR, whose full-wave band models met the prediction standards for predicting the content of potassium in pear pulp, were optimized to extract the characteristic wavelengths. Then, GA extracted 118 and 114 characteristic band points from the original spectral curve and the spectral curve preprocessed by SG, respectively. As shown in Figure 10, on the original spectral curve, the extracted characteristic wavelengths were densely distributed in the range of 900–1100 nm, 1200–1300 nm and 1500–1680 nm. Meanwhile, on the spectral curve after SG pretreatment, the extracted characteristic wavelengths were most densely distributed in the range of 1020–1150 nm and 1430–1650 nm. It can be inferred that the response of the compounds related to the content of potassium in pear pulp on the spectral curve was mostly concentrated in the range of 1000–1100 nm and 1500–1650 nm.

(3) Modelling of characteristic wavelengths

**Figure 11:**
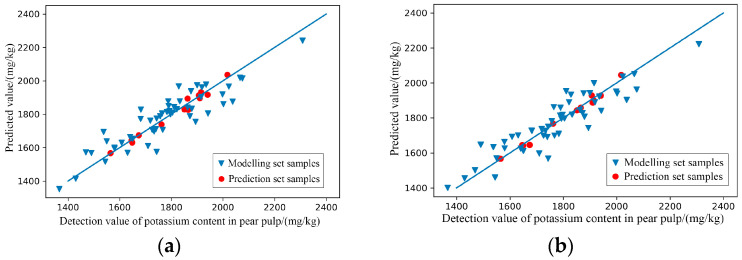
Scatter plots of models’ prediction results of the content of potassium in pear pulp samples. (**a**) Raw-GA-PLSR; (**b**) SG-GA-PLSR.

According to the results shown in Table 5, after GA treatment, the prediction abilities of RAW-PLSR and SG-PLSR were both greatly improved, and the model levels also rose from level B to level A. The scatter plots of the prediction results are shown in Figure 11, and they more intuitively show that both Raw-GA-PLSR and SG-GA-PLSR can more accurately predict the content of potassium in the pulp of ‘Huangguan’ pears.

(4) Modelling effect evaluation

After discussion and analysis, SG-GA-PLSR had the best performance in predicting the content of potassium in pear pulp, with R^2^ = 0.834 and RPD = 1.810 for the modelling set and R^2^ = 0.985 and RPD = 5.833 for the prediction set.

### 3.5. Modelling and Prediction Analysis of the Content of Mineral Elements in Pear Peel

Table 6 shows the comparison of parameters R^2^ and RPD of each prediction model for calcium, boron and potassium content in pear peel based on a full-wave band.

#### 3.5.1. Modelling and Prediction Analysis of the Content of Calcium in Pear Peel

(1) Full-band based modelling

Among the 13 PLSR models established, the predicting level of RAW-PLSR, SNV-PLSR, MSC-PLSR, SG-PLSR, LG-PLSR and SG+SNV-PLSR were all B. Meanwhile, although the R^2^ and RPD of the modelling sets of SNV-PLSR, MSC-PLSR and SG+SNV-PLSR decreased slightly compared with the original models, the R^2^ and RPD of the prediction sets of these three models were improved, which means that they may have better abilities to predict the content of calcium in the peel of ‘Huangguan’ pears. Furthermore, among the 13 established GBRT models, although the predictive effects of MSC-GBRT and SG+MSC-GBRT were outstanding at the modelling sets, their R^2^ and RPD of the prediction sets did not meet the predictive standards.

(2) Characteristic wavelength extraction

**Figure 12:**
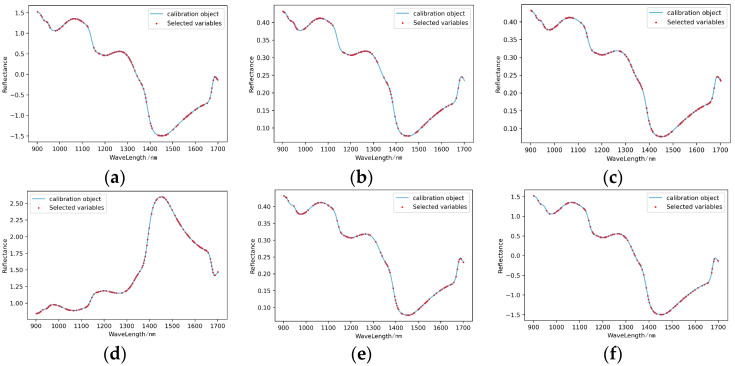
Characteristic wavelengths’ distribution of prediction models for the content of calcium in pear peel. (**a**) SNV-GA-PLSR; (**b**) MSC-GA-PLSR; (**c**) SG-GA-PLSR; (**d**) LG-GA-PLSR; (**e**) SG+MSC-GA-PLSR; (**f**) SG+SNV-GA-PLSR.

As shown in Figure 12, GA extracted 113, 109, 118, 136, 105 and 104 characteristic wavelengths from the spectral curves processed by SNV, MSC, SG, LG, SG+MSC and SG+SNV, respectively. Firstly, the characteristic wavelengths extracted from the spectral curve processed by SNV were roughly concentrated at 1000–1100 nm, 1200–1350 nm and 1550–1680 nm; secondly, the characteristic wavelengths extracted from the spectral curve points processed by MSC were concentrated at 900–1100 nm, 1200–1300 nm and 1500–1650 nm; thirdly, the characteristic wavelengths extracted from the spectral curve points processed by SG were concentrated at 900–1080 nm, 1150–1380 nm and 1500–1650 nm; fourthly, the characteristic wavelengths extracted from the spectral curve points processed by LG were concentrated at 900–1350 nm and 1500–1600 nm; fifthly, the characteristic wavelengths extracted from the spectral curve points processed by SG+MSC were concentrated at 1000–1100 nm and 1450–1650 nm; and finally, the characteristic wavelengths extracted from the spectral curve points processed by SG+SNV were concentrated at 1000–1300 nm and 1400–1680 nm. Overall, the characteristic wavelengths extracted from the six spectral curves were mostly concentrated in 1000–1100 nm, 1200–1300 nm and 1500–1600 nm, indicating that the information reflected by the organic matters related to the content of calcium in the peel was mostly concentrated on these three bands.

(3) Modelling of characteristic wavelengths

**Figure 13:**
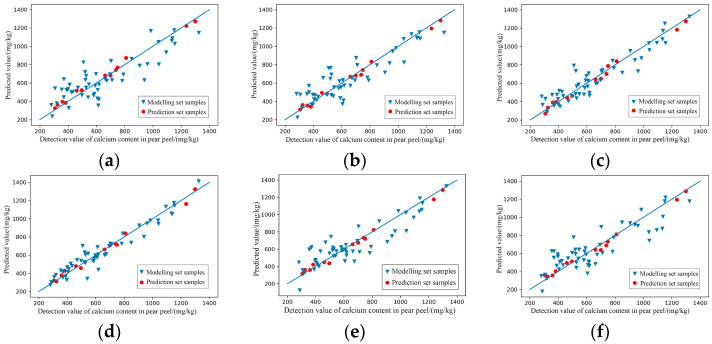
Scatter plots of models’ prediction results of the content of calcium in pear peel samples. (**a**) SNV-GA-PLSR; (**b**) MSC-GA-PLSR; (**c**) SG-GA-PLSR; (**d**) LG-GA-PLSR; (**e**) SG+MSC-GA-PLSR; (**f**) SG+SNV-GA-PLSR.

After the GA treatment, the prediction abilities of the six models for predicting the content of calcium in pear peel were improved. As the results shown in Table 7 and Figure 13, the prediction effects of the modelling set and prediction set of MSC-GA-PLSR and SG+MSC-GA-PLSR all reached level A. Although the prediction sets of SNV-GA-PLSR, SG-GA-PLSR, LG-GA-PLSR and SG+SNV-GA-PLSR met level A, the level of the modelling sets remained at level B; moreover, the R^2^ and RPD of the modelling sets were slightly lower than those of the model without feature processing. Firstly, this may have been because, in the process of feature wavelength extraction, the eliminated feature wavelengths may have contained useful information that was closely related to later modelling, and the lack of this part of the information had a certain impact on the accuracy of the models. Secondly, it may have also been related to the data structure characteristics of the original modelling sets.

(4) Modelling effect evaluation

Through comparative analysis, LG-GA-PLSR was the best model for predicting the content of calcium in pear peels, with R^2^ = 0.93 and RPD = 2.671 in the modelling set and R^2^ = 0.989 and RPD = 6.951 in the prediction set, which also met level A.

#### 3.5.2. Modelling and Prediction Analysis of the Content of Boron in Pear Peel

(1) Full-band based modelling

Among the 12 PLSR models based on the spectrum after pretreatment, SNV-PLSR, MSC-PLSR, SG+MSC-PLSR and SG+SNV-PLSR significantly improved the R^2^ and RPD of the prediction sets compared with RAW-PLSR, and reached level B. Among the 13 GBRT models for predicting the content of boron in pear peels, although the modelling sets of FD-GBRT, SD-GBRT, LG-GBRT, SG+MSC+FD-GBRT, SG+MSC+SD-GBRT and SG+SNV performed well, with R^2^ exceeding 0.8 and RPD exceeding 1.4, their prediction sets did not meet the basic prediction criteria.

(2) Characteristic wavelength extraction

**Figure 14:**
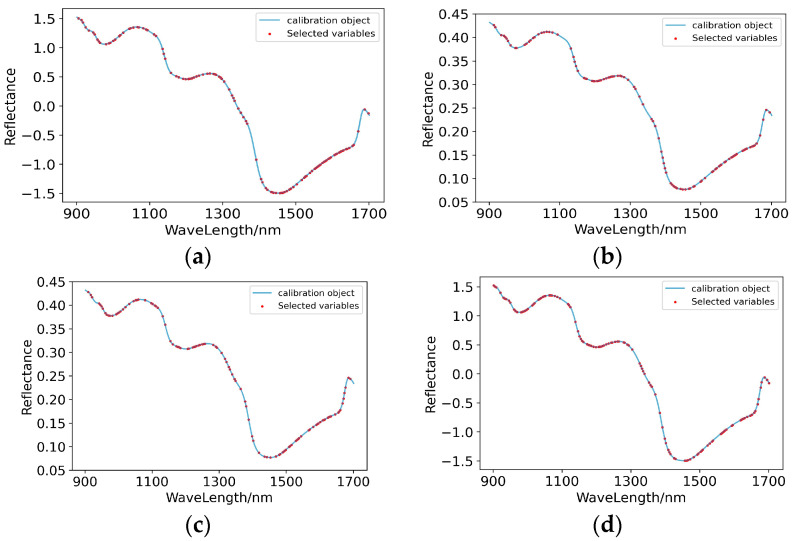
Characteristic wavelengths’ distribution of prediction models for the content of boron in pear peel. (**a**) SNV-GA-PLSR; (**b**) MSC-GA-PLSR; (**c**) SG+MSC-GA-PLSR; (**d**) SG+SNV-GA-PLSR.

Among the 26 established full-wave band models, SNV-PLSR, MSC-PLSR, SG+MSC-PLSR and SG+SNV-PLSR, which initially met the prediction standards, were further extracted with characteristic wavelengths. GA extracted 116, 109, 108 and 124 characteristic wavelengths from the spectral curves preprocessed by SNV, MSC, SG+MSC and SG+SNV, respectively. It can be seen from Figure 14 that on the spectral curve after SNV, the extracted characteristic wavelengths were most densely distributed in the range of 900–1070 nm, 1200–1350 nm and 1450–1650 nm; on the spectral curve after MSC, the extracted characteristic wavelengths were most densely distributed in the range of 910–1090 nm, 1200–1300 nm and 1400–1680 nm; on the spectral curve after SG+MSC, the extracted characteristic wavelengths were most densely distributed in the range of 940–1060 nm, 1130–1260 nm and 1490–1680 nm; on the spectral curve after SG+SNV, the extracted characteristic wavelengths were most densely distributed in the range of 960–1090 nm, 1150–1300 nm and 1450–1680 nm. The dense distribution areas of the extracted characteristic wavelengths were similar, which indicates that the compounds with a great correlation with the content of boron in the pear peels mainly responded in the range of 900–1050 nm, 1200–1300 nm and 1500–1650 nm.

(3) Modelling of characteristic wavelengths

**Figure 15:**
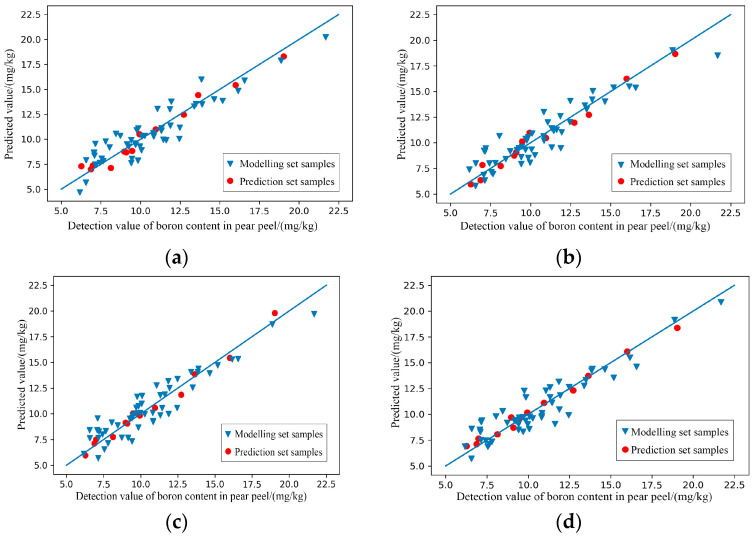
Scatter plots of models’ prediction results of the content of boron in pear peel samples. (**a**) SNV-GA-PLSR; (**b**) MSC-GA-PLSR; (**c**) SG+MSC-GA-PLSR; (**d**) SG+SNV-GA-PLSR.

As the results shown in Table 8 and Figure 15, after the GA treatment, the performances of SNV-GA-PLSR, MSC-GA-PLSR, SG+MSC-GA-PLSR and SG+SNV-GA-PLSR for predicting the content of boron in the pear peel were improved to varying degrees. The scatter plots of the prediction results shown in Figure 15 further intuitively show the predictive effects of the models.

(4) Modelling effect evaluation

Through comparative analysis, SG+MSC-GA-PLSR had the best effect on predicting the content of boron in pear peels, with the R^2^ of the modelling set = 0.870 and RPD = 2.027, and the R^2^ of the prediction set = 0.984 and RPD = 5.598.

#### 3.5.3. Modelling and Prediction Analysis of the Content of Potassium in Pear Peel

(1) Full-band based modelling

As shown in Table 5, among the 13 PLSR models established for predicting the content of potassium in pear peels, SNV-PLSR and SG+SNV-PLSR met the predictive standards, and the R^2^ and RPD of the SG+SNV-PLSR prediction set were slightly higher than those of the SNV-PLSR prediction set. This may be because the SG+SNV pretreatment further smoothed the curve and improved the modelling accuracy based on the SNV. At the same time, among the 13 GBRT models, SNV-GBRT, MSC-GBRT, SD-GBRT, SG+MSC-GBRT, SG+SNV-GBRT, SG+MSC+SD-GBRT and SG+SNV+SD-GBRT failed. The remaining six GBRT models belonged to level C and could not be used to predict the content of potassium in the peel of ‘Huangguan’ pears.

(2) Characteristic wavelength extraction

**Figure 16:**
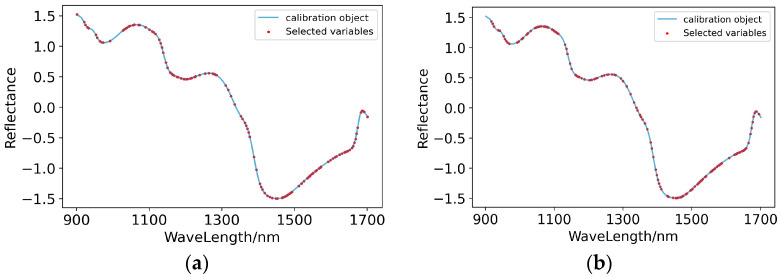
Characteristic wavelengths’ distribution of prediction models for the content of potassium in pear peel. (**a**) SNV-GA-PLSR; (**b**) SG+SNV-GA-PLSR.

SNV-PLSR and SG+SNV-PLSR, which preliminarily met the prediction standards, were processed by extracting the characteristic wavelengths on the spectral curves. GA extracted 122 and 133 characteristic wavelengths from the spectral curves pretreated by SNV and SG+SNV, respectively. As shown in Figure 16, on the spectral curve pretreated by SNV, the extracted characteristic wavelengths were mainly concentrated in 1160–1280 nm and 1400–1650 nm; on the spectral curve pretreated by SG+SNV, the extracted characteristic wavelengths were mainly concentrated at 1050–1110 nm, 1200–1300 nm and 1450–1680 nm. Therefore, the response intervals of the compounds related to the content of potassium in the pear peel on the spectral curve were mainly 1200–1300 nm and 1450–1650 nm.

(3) Modelling of characteristic wavelengths

**Figure 17:**
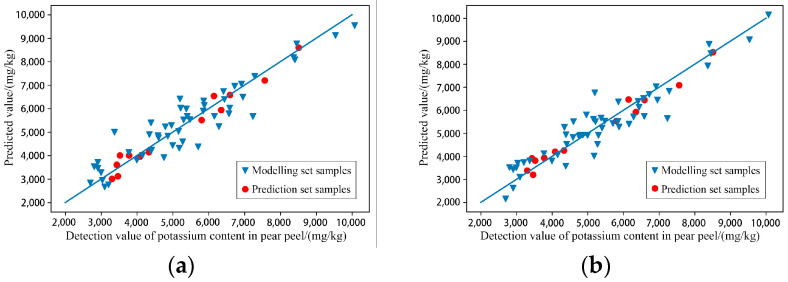
Scatter plots of models’ prediction results of the content of potassium in pear peel samples. (**a**) SNV-GA-PLSR; (**b**) SG+SNV-GA-PLSR.

It can be seen from Table 9 and Figure 17 that the prediction ability of the model built after GA processing was improved. Although the R^2^ and RPD of the SNV-GA-PLSR and SG+SNV-GA-PLSR modelling sets were slightly decreased, the R^2^ and RPD of their prediction sets were higher than those before feature extraction processing, and the prediction level of the model increased to level A.

(4) Modelling effect evaluation

From the discussion and comparison above, we can see that SG+SNV-GA-PLSR had the best ability to predict the content of potassium in pear pulp with an R^2^ = 0.888 and RPD = 2.175 for the modelling set, and an R^2^ = 0.973 and RPD = 4.294 for the prediction set.

## 4. Discussion

Previous studies have pointed out that the brown spots that seriously affect the appearance quality of ‘Huangguan’ pears are caused by physiological disease, [38] and are largely related to the content and proportion of mineral elements in the pulp and peel of pears. In previous studies, the mineral elements calcium, boron and potassium were proved to be important factors affecting the appearance quality of ‘Huangguan’ pears, and the content of mineral elements in the pulp and peel of diseased pears may have a certain degree of correlation, which will show a different appearance with the severity of the disease [6]. The timely detection of the content of calcium, boron and potassium in fruits and the adjustment of fertilization and bagging based on the detected results are of great significance to the improvement of fruit quality in the later stage. Compared with the traditional laboratorial and expensive physical and chemical detection methods, near-infrared spectroscopy has been applied to the study of soluble solids, titratable acids and the mineral element content of pears for its several potential advantages [20,21,22,23]. Therefore, in this paper, which is based on the visible-near-infrared (900–1700 nm) spectroscopy technology, the ‘NIR-S-G1′ micro spectrometer was used to collect spectral data, and combined with 12 pretreatment methods, the content of calcium, boron and potassium in ‘Huangguan’ pears’ fruit (including peel and pulp) were predicted by PLSR and GBRT. For the models that met the prediction standard, GA was used to extract the characteristic wavelengths of its original spectral curve to further optimize the models.

The full-wave band modelling results showed that the overall prediction abilities of the PLSR models were stronger than those of the GBRT models. The GBRT integration algorithm combines the advantages of many algorithms, but its performance in processing high-dimensional data with small samples was not as good as that of the traditional single regression model PLSR. This is roughly the same as what was found in previous studies [39,40,41], but there are also differences. Relatively speaking, the overall prediction effects of the models with feature extraction by the genetic algorithm were better than those of the full-band models without feature extraction. Although the specific characteristic wavelengths extracted from each model were not the same, the distribution ranges of the extracted characteristic wavelengths on the original spectral curve were similar, which may have been due to the similarity of the compounds corresponding to the corresponding mineral elements, resulting in the similar chemical bond vibration laws contained in the corresponding compounds. Previous studies have shown that the chemical components in the pulp and peel of pears are complex, and the main chemical components in pears include flavonoids, triterpenes, phenolic acids, etc. Generally, the peel of a pear contains more phenolic acids, flavonoids and triterpenes than the pulp. The presentation of the spectral curves also corresponds to the frequency doubling and frequency combining information of different chemical bond vibrations. In the range of 900–1700 nm, some chemical bonds with a strong saturation activity, especially the X-H bond, and some other chemical bonds are also active in this range, such as the C=O bond of ester and the C=N bond of amine. This paper may provide some theoretical basis for the study of pear fruit characteristic wavelength modelling.

The results of this study showed that the best models for predicting the content of calcium in the pulp and peel of ‘Huangguan’ pears were RAW-GA-PLSR (the R^2^ and RPD of the modelling set were 0.898 and 2.271, respectively, and the R^2^ and RPD of the prediction set were 0.987 and 6.110, respectively) and LG-GA-PLSR (the R^2^ and RPD of the modelling set were 0.93 and 2.671, respectively, and the R^2^ and RPD of the prediction set were 0.989 and 6.951, respectively). The prediction ability of LG-GA-PLSR was slightly stronger than RAW-GA-PLSR, which may have occurred because the compounds related to the content of calcium in pear peels are more sensitive in the range of 900–1700 nm. Secondly, the best models for predicting the content of boron in the pulp and peel of ‘Huangguan’ pears were SG-GA-PLSR (the R^2^ and RPD of the modelling set were 0.846 and 1.878, respectively, and the R^2^ and RPD of the prediction set were 0.986 and 6.033, respectively) and SG+MSC-GA-PLSR (the R^2^ and RPD of the modelling set were 0.870 and 2.027, respectively, and the R^2^ and RPD of the prediction set were 0.984 and 5.598, respectively). The prediction effect of SG-GA-PLSR was slightly stronger than that of SG+MSC-GA-PLSR, which may have occurred due to the pretreatment method, or because the compounds related to the content of boron in the pulp have a stronger response within the spectral range determined in this paper. Thirdly, the best models for predicting the content of potassium in the pulp and peel of ‘Huangguan’ pears were SG-GA-PLSR (the R^2^ and RPD of the modelling set were 0.834 and 1.810, respectively, and the R^2^ and RPD of the prediction set were 0.985 and 5.833, respectively) and SG+SNV-GA-PLSR (the R^2^ and RPD of the modelling set were 0.888 and 2.175, respectively, and the R^2^ and RPD of the prediction set were 0.973 and 4.294, respectively).

## 5. Conclusions

From the discussion above, it can be concluded that near-infrared spectroscopy can be used in the effectively and accurately nondestructive prediction of calcium, boron and potassium in the pulp and peel of ‘Huangguan’ pears. Furthermore, the precision of the mineral element content in the pulp was slightly higher than that in the peel, so the occurrence of the skin browning of ‘Huangguan’ pears can be more accurately predicted. The research in this paper provides a theoretical basis for the nondestructive prediction of the element content affecting the appearance quality of ‘Huangguan’ pears. The next stage is the validation of methods, which refers to the demonstration of the suitability of the method by analyzing random samples, to further enrich the modeling samples from different dimensions and to optimize the model, by combining it with artificial intelligence to implement the application of the research results in the field.

## Figures and Tables

**Table 1:** Overview of mineral elements’ content detection.

	Calcium in Pulp	Boron in Pulp	Potassium in Pulp	Calcium in Peel	Boron in Peel	Potassium in Peel
Maximum (mg/kg)	40.5	7.865	2308	1324.85	21.675	10,073
Minimum (mg/kg)	14	2.115	1365.8	281.15	6.175	2691.85
Average (mg/kg)	26.687	3.995	1783.7	653.44	10.646	5323.39

**Table 2:** Comparison of the prediction and modelling sets for the content of calcium, boron and potassium in pear pulp based on full-wave band.

Models	Prediction Results for the Content of Calcium in Pear Pulp				Prediction Results for the Content of Boron in Pear Pulp				Prediction Results for the Content of Potassium in Pear Pulp			
	R^2^ of Modelling Set	RPD of Modelling Set	R^2^ of Prediction Set	RPD of Modelling Set	R^2^ of Modelling Set	RPD of Modelling Set	R^2^ of Prediction Set	RPD of Prediction Set	R^2^ of Modelling Set	RPD of Modelling Set	R^2^ of Prediction Set	RPD of Prediction Set
Raw-PLSR	0.92	2.574	0.716	1.431	0.904	2.344	0.734	1.472	0.86	1.97	0.767	1.557
SNV-PLSR	0.932	2.768	0.684	1.371	0.516	1.167	0.495	1.151	0.97	4.452	0.382	1.082
FD-PLSR	0.76	1.535	0.348	1.066	0.207	1.022	0.212	1.023	0.99	7.089	0.141	1.01
MSC-PLSR	0.936	2.832	0.651	1.317	0.568	1.215	0.492	1.149	0.99	7.089	0.141	1.01
SD-PLSR	0.694	1.388	0.304	1.05	0.712	1.424	-	-	0.97	4.452	0.382	1.082
SG-PLSR	0.921	2.574	0.717	1.433	0.904	2.344	0.735	1.475	0.86	1.973	0.767	1.558
LG-PLSR	0.991	7.376	0.582	1.23	0.871	2.036	0.497	1.152	0.86	1.975	0.658	1.328
SG+MSC-PLSR	0.936	2.832	0.651	1.317	0.568	1.215	0.492	1.149	0.99	7.089	0.141	1.01
SG+SNV-PLSR	0.932	2.768	0.684	1.371	0.516	1.167	0.495	1.151	1	-	0.31	1.053
SG+MSC+FD-PLSR	0.705	1.409	0.338	1.063	0.187	1.018	0.216	1.024	0.98	4.946	0.62	1.275
SG+MSC+SD-PLSR	0.676	1.356	0.301	1.048	0.705	1.41	-	-	0.74	1.487	0.432	1.109
SG+SNV+FD-PLSR	0.707	1.415	0.346	1.066	0.191	1.019	0.218	1.025	0.98	4.961	0.625	1.282
SG+SNV+SD-PLSR	0.678	1.36	0.299	1.048	0.705	1.411	-	-	0.98	4.961	0.625	1.282
Raw-GBRT	0.982	5.318	0.511	1.163	0.562	1.209	0.278	1.038	0.012	1	-	-
SNV-GBRT	0.979	4.938	-	0	0.975	4.477	0.448	1.119	0.014	1	-	-
FD-GBRT	0.99	7.089	0.411	1.097	0.989	7.063	0.131	1.009	0.017	1	-	-
MSC-GBRT	0.419	1.101	-	0	0.908	2.392	0.539	1.187	0.015	1	-	-
SD-GBRT	0.961	3.617	-	0	0.995	10.28	0.483	1.142	0.999	7.091	0.05	1.001
SG-GBRT	0.982	5.318	0.512	1.164	0.562	1.209	0.268	1.038	0.998	15.819	0.609	1.261
LG-GBRT	0.956	3.426	0.441	1.114	0.432	1.109	0.264	1.037	0.979	4.85	0.547	1.195
SG+MSC-GBRT	0.016	1	-	0	0.908	2.392	0.539	1.187	0.015	1	-	-
SG+SNV-GBRT	0.979	4.938	-	0	0.975	4.477	0.448	1.119	0.014	1	-	-
SG+MSC+FD-GBRT	0.983	5.385	0.6	1.244	0.949	3.165	0.436	1.111	0.016	1	-	-
SG+MSC+SD-GBRT	0.999	7.089	-	0	0.858	1.948	0.276	1.04	0.989	6.705	0.293	1.046
SG+SNV+FD-GBRT	0.999	7.09	0.362	1.073	0.987	6.193	0.222	1.026	0.016	1	-	-
SG+SNV+SD-GBRT	0.996	10.659	-	0	0.858	1.948	0.276	1.04	0.989	6.705	0.293	1.046

Note: “-” means the value here is invalid, the same below.

**Table 3:** Modelling prediction results of the content of calcium in pear pulp based on the selected characteristic wavelengths by using GA.

Models	Modelling Set		Prediction Set		Level of Models
	R^2^	RPD	R^2^	RPD	
Raw-GA-PLSR	0.898	2.271	0.987	6.110	A
SG-GA-PLSR	0.895	2.246	0.973	4.325	A

**Table 4:** Modelling prediction results of the content of boron in pear pulp based on the selected characteristic wavelengths.

Methods of Extracting Characteristic Wavelengths	Models	Modelling Set		Prediction Set		Level of Models
		R^2^	RPD	R^2^	RPD	
GA	Raw-GA-PLSR	0.768	1.560	0.984	5.560	A
	SG-GA-PLSR	0.846	1.878	0.986	6.033	A

**Table 5:** Modelling prediction results of the content of potassium in pear pulp based on the selected characteristic wavelengths.

Methods of Extracting Characteristic Wavelengths	Models	Modelling Set		Prediction Set		Level of Models
		R^2^	RPD	R^2^	RPD	
GA	Raw-GA-PLSR	0.823	1.760	0.978	4.756	A
	SG-GA-PLSR	0.834	1.810	0.985	5.833	A

**Table 6:** Comparison of the prediction and modelling sets for the content of calcium, boron and potassium in pear peel based on full-wave band.

Models	Prediction Results for the Content of Calcium in Pear Peel				Prediction Results for the Content of Boron in Pear Peel				Prediction Results for the Content of Potassium in Pear Peel			
	R^2^ of Modelling Set	RPD of Modelling Set	R^2^ of Prediction Set	RPD of Modelling Set	R^2^ of Modelling Set	RPD of Modelling Set	R^2^ of Prediction Set	RPD of Prediction Set	R^2^ of Modelling Set	RPD of Modelling Set	R^2^ of Prediction Set	RPD of Prediction Set
Raw-PLSR	0.81	1.705	0.708	1.416	0.829	1.79	0.186	1.018	0.097	1.005	0.12	1.007
SNV-PLSR	0.76	1.53	0.811	1.711	0.897	2.257	0.707	1.414	0.895	2.241	0.756	1.529
FD-PLSR	0.15	1.011	0.456	1.124	0.996	11.154	0.155	1.012	0.512	1.164	0.274	1.04
MSC-PLSR	0.78	1.603	0.78	1.599	0.942	2.989	0.701	1.402	0.718	1.436	0.472	1.135
SD-PLSR	0.61	1.258	0.12	1.007	0.665	1.338	0.038	1.001	0.669	1.345	0.095	1.005
SG-PLSR	0.91	2.458	0.808	1.696	0.829	1.79	0.186	1.018	0.097	1.005	0.12	1.007
LG-PLSR	0.96	3.437	0.796	1.651	0.857	1.94	0.221	1.025	0.521	1.171	0.117	1.007
SG+MSC-PLSR	0.78	1.603	0.78	1.599	0.942	2.989	0.701	1.402	0.718	1.436	0.472	1.135
SG+SNV-PLSR	0.76	1.53	0.812	1.713	0.897	2.257	0.709	1.415	0.895	2.241	0.757	1.531
SG+MSC+FD-PLSR	0.14	1.01	0.421	1.103	0.833	1.808	0.145	1.011	0.825	1.771	0.036	1.001
SG+MSC+SD-PLSR	0.6	1.248	0.104	1.005	0.848	1.885	0.006	1	0.999	7.089	0.064	1.002
SG+SNV+FD-PLSR	0.14	1.01	0.421	1.102	0.849	1.895	0.148	1.011	0.582	1.23	0.041	1.001
SG+SNV+SD-PLSR	0.6	1.248	0.104	1.005	0.849	1.895	0.148	1.011	0.999	7.089	0.064	1.002
Raw-GBRT	0.99	7.16	0.423	1.104	0.54	1.188	0.075	1.003	0.884	2.143	0.24	1.03
SNV-GBRT	0.99	15.802	0.431	1.295	0.103	1.005	−0.05	-	0.258	1.035	−0.007	-
FD-GBRT	0.99	7.089	0.499	1.154	0.827	1.777	0.345	1.065	0.711	1.422	0.065	1.002
MSC-GBRT	0.99	7.089	0.512	1.164	0.014	1	−0.007	-	0.411	1.097	−0.057	-
SD-GBRT	0.93	2.691	0.411	1.097	0.991	7.532	0.236	1.029	0.063	1.002	−0.037	-
SG-GBRT	0.99	7.089	0.423	1.104	0.54	1.188	0.075	1.003	0.884	2.143	0.24	1.03
LG-GBRT	0.99	7.089	0.48	1.14	0.826	1.776	0.039	1.001	0.884	2.143	0.245	1.031
SG+MSC-GBRT	0.99	7.089	0.512	1.164	0.014	1	−0.007	-	0.073	1.003	−0.023	-
SG+SNV-GBRT	0.99	7.089	0.431	1.108	0.103	1.005	−0.05	-	0.258	1.035	−0.007	-
SG+MSC+FD-GBRT	0.99	18.101	0.509	1.162	0.931	2.747	0.071	1.003	0.913	2.447	0.263	1.037
SG+MSC+SD-GBRT	0.99	7.089	0.351	1.068	0.971	4.179	0.232	1.028	0.929	2.693	−0.006	-
SG+SNV+FD-GBRT	0.99	7.089	0.498	1.153	0.998	15.819	0.135	1.009	0.681	1.366	0.168	1.014
SG+SNV+SD-GBRT	0.99	7.089	0.367	1.113	0.915	2.483	0.167	1.014	0.205	1.022	−0.011	-

**Table 7:** Modelling prediction results of the content of calcium in pear peel based on the selected characteristic wavelengths.

Methods of Extracting Characteristic Wavelengths	Models	Modelling Set		Predicting Set		Level of Models
		R^2^	RPD	R^2^	RPD	
GA	SNV-GA-PLSR	0.73	1.454	0.989	6.837	A
	MSC-GA-PLSR	0.80	1.675	0.992	8.119	A
	SG-GA-PLSR	0.87	2.054	0.989	6.672	A
	LG-GA-PLSR	0.93	2.671	0.989	6.951	A
	SG+MSC-GA-PLSR	0.79	1.654	0.991	7.282	A
	SG+SNV-GA-PLSR	0.74	1.481	0.989	6.858	A

**Table 8:** Modelling prediction results of the content of boron in pear peel based on the selected characteristic wavelengths.

Methods of Extracting Characteristic Wavelengths	Models	Modelling Set		Predicting Set		Level of Models
		R^2^	RPD	R^2^	RPD	
GA	SNV-GA-PLSR	0.855	1.926	0.973	4.301	A
	MSC-GA-PLSR	0.860	1.962	0.973	4.344	A
	SG+MSC-GA-PLSR	0.870	2.027	0.984	5.598	A
	SG+SNV-GA-PLSR	0.859	1.953	0.986	6.077	A

**Table 9:** Modelling prediction results of the content of potassium in pear peel based on the selected characteristic wavelengths.

Methods of Extracting Characteristic Wavelengths	Models	Modelling Set		Prediction Set		Level of Models
		R^2^	RPD	R^2^	RPD	
GA	SNV-GA-PLSR	0.873	2.052	0.971	4.148	A
	SG+SNV-GA-PLSR	0.888	2.175	0.973	4.294	A

## Data Availability

The datasets generated for this study are available on request to the corresponding author.
